# Midwifery continuity of care versus standard maternity care for women at increased risk of preterm birth: A hybrid implementation–effectiveness, randomised controlled pilot trial in the UK

**DOI:** 10.1371/journal.pmed.1003350

**Published:** 2020-10-06

**Authors:** Cristina Fernandez Turienzo, Debra Bick, Annette L. Briley, Mary Bollard, Kirstie Coxon, Pauline Cross, Sergio A. Silverio, Claire Singh, Paul T. Seed, Rachel M. Tribe, Andrew H. Shennan, Jane Sandall, on behalf of the POPPIE Pilot Collaborative Group

**Affiliations:** 1 Department of Women and Children’s Health, Faculty of Life Science and Medicine, King’s College London, London, United Kingdom; 2 Warwick Clinical Trials Unit, Warwick Medical School, University of Warwick, Coventry, United Kingdom; 3 Caring Futures Institute, Flinders University, Adelaide, Australia; 4 Maternity Services, Lewisham and Greenwich NHS Trust, London, United Kingdom; 5 Department of Midwifery, Kingston University and St. George’s, University of London, United Kingdom; 6 Department of Public Health, London Borough of Lewisham, London, United Kingdom; Burnet Institute, AUSTRALIA

## Abstract

**Background:**

Midwifery continuity of care is the only health system intervention shown to reduce preterm birth (PTB) and improve perinatal survival, but no trial evidence exists for women with identified risk factors for PTB. We aimed to assess feasibility, fidelity, and clinical outcomes of a model of midwifery continuity of care linked with a specialist obstetric clinic for women considered at increased risk for PTB.

**Methods and findings:**

We conducted a hybrid implementation–effectiveness, randomised, controlled, unblinded, parallel-group pilot trial at an inner-city maternity service in London (UK), in which pregnant women identified at increased risk of PTB were randomly assigned (1:1) to either midwifery continuity of antenatal, intrapartum, and postnatal care (Pilot study Of midwifery Practice in Preterm birth Including women’s Experiences [POPPIE] group) or standard care group (maternity care by different midwives working in designated clinical areas). Pregnant women attending for antenatal care at less than 24 weeks' gestation were eligible if they fulfilled one or more of the following criteria: previous cervical surgery, cerclage, premature rupture of membranes, PTB, or late miscarriage; previous short cervix or short cervix this pregnancy; or uterine abnormality and/or current smoker of tobacco. Feasibility outcomes included eligibility, recruitment and attrition rates, and fidelity of the model. The primary outcome was a composite of appropriate and timely interventions for the prevention and/or management of preterm labour and birth. We analysed by intention to treat. Between 9 May 2017 and 30 September 2018, 334 women were recruited; 169 women were allocated to the POPPIE group and 165 to the standard group. Mean maternal age was 31 years; 32% of the women were from Black, Asian, and ethnic minority groups; 70% were in employment; and 46% had a university degree. Nearly 70% of women lived in areas of social deprivation. More than a quarter of women had at least one pre-existing medical condition and multiple risk factors for PTB. More than 75% of antenatal and postnatal visits were provided by a named/partner midwife, and a midwife from the POPPIE team was present at 80% of births. The incidence of the primary composite outcome showed no statistically significant difference between groups (POPPIE group 83.3% versus standard group 84.7%; risk ratio 0.98 [95% confidence interval (CI) 0.90 to 1.08]; p = 0.742). Infants in the POPPIE group were significantly more likely to have skin-to-skin contact after birth, to have it for a longer time, and to breastfeed immediately after birth and at hospital discharge. There were no differences in other secondary outcomes. The number of serious adverse events was similar in both groups and unrelated to the intervention (POPPIE group 6 versus standard group 5). Limitations of this study included the limited power and the nonmasking of group allocation; however, study assignment was masked to the statistician and researchers who analysed the data.

**Conclusions:**

In this study, we found that it is feasible to set up and achieve fidelity of a model of midwifery continuity of care linked with specialist obstetric care for women at increased risk of PTB in an inner-city maternity service in London (UK), but there is no impact on most outcomes for this population group. Larger appropriately powered trials are needed, including in other settings, to evaluate the impact of relational continuity and hypothesised mechanisms of effect based on increased trust and engagement, improved care coordination, and earlier referral on disadvantaged communities, including women with complex social factors and social vulnerability.

**Trial registration:**

We prospectively registered the pilot trial on the UK Clinical Research Network Portfolio Database (ID number: 31951, 24 April 2017). We registered the trial on the International Standard Randomised Controlled Trial Number (ISRCTN) (Number: 37733900, 21 August 2017) and before trial recruitment was completed (30 September 2018) when informed that prospective registration for a pilot trial was also required in a primary clinical trial registry recognised by WHO and the International Committee of Medical Journal Editors (ICMJE). The protocol as registered and published has remained unchanged, and the analysis conforms to the original plan.

## Abstract 2

Cristina Fernandez Turienzo and colleagues assess a model to improve continuity of the antenatal, intrapartum, and postnatal care for women at increased risk for preterm birth.

## Author summary

**Why was this study done?:**

- Models of midwifery continuity of care models are recommended in international guidance and at the heart of maternity policy in the United Kingdom, where there is a recommendation to scale up continuity models on the grounds of improving high-quality and safe maternity care.
- These models, in which the same midwife or team of midwives provide care during pregnancy, birth, and the early parenting period, are the only health system intervention shown to reduce both preterm birth and improve perinatal survival among women at low and mixed risk of complications. However, no trial evidence exists for women with identified risk factors for preterm birth.

**What did the researchers do and find?:**

- We did a hybrid randomised controlled pilot trial to assess implementation process measures and clinical outcomes of a model of continuity of midwifery care with access to a specialist obstetric clinic for women considered to be at increased risk for preterm birth.
- We found it is feasible to set up and maintain fidelity to this model of care in a UK inner-city maternity service in London. There was no difference in most clinical outcomes, although these may not have been sufficiently powered.

**What do these findings mean?:**

- Although this is a pilot trial, the lack of trend for most outcomes indicates that for this population of women at increased risk of preterm birth, this model of care does not show promise of impact to justify a larger trial with these outcomes in this population.
- Further research should investigate, including in other settings, whether hypothesised mechanisms based on increased trust and engagement, improved care coordination and earlier referral, and continuity of midwife care can impact disadvantaged communities, including women with complex social factors and social vulnerability for which there is little evidence.

## Introduction

Preterm birth (PTB) is defined as any birth that occurs before 37 completed weeks’ gestation [1]. One in 10 babies worldwide are born early every year, and over a million die from complications associated with their prematurity [2]. Many of those who do survive are particularly vulnerable to significant disabilities and health problems throughout their lives (e.g., learning disabilities, hearing and visual impairments, chronic lung disease), which results in a major burden for families, societies, and healthcare systems [3]. Many efforts have been taken to reduce the prevalence of PTB, improve clinical management, and decrease neonatal morbidity and mortality, but early births continue to rise in most countries where reliable data are available [4].

Nearly two-thirds of PTBs are classified as spontaneous, due to spontaneous onset of labour or preterm prelabour rupture of membranes (PPROM), with the remainder classified as iatrogenic or provider-initiated (early induction or cesarean section for a fetal or maternal reasons) [5]. A wide range of sociodemographic, biological, nutritional, psychological, and environmental factors have been associated with spontaneous PTB, and its complex and multifactorial nature challenges the understanding of the causal pathways leading to PTB [5–6]. This consequently limits the development and implementation of appropriate clinical interventions and public health prevention strategies, including specific maternity care packages designed to address prematurity.

A recent Cochrane review found that models of midwifery continuity of care for childbearing women were the only health service and system intervention associated with both a reduction in PTBs and improvements in perinatal survival [7]. Women who received these care models were, on average, 24% less likely to have a PTB and 19% less likely to lose their babies before 24 weeks’ gestation [8]. These models involve the planning, organisation, and delivery of comprehensive maternity care by 1 named midwife or a small team of midwives given to a woman during her pregnancy, birth, and early parenting periods. Midwives work in partnership with the woman and are the primary professionals responsible for the assessment of needs, care planning, referral to other professionals, and ensuring the coordination of services [8]. However, models of care are complex interventions and require theoretical modelling of the relationships between processes and outcomes. It is unclear whether the pathway of influence that produces these outcomes in a low-risk or geographical population of women would have the same impact in women who have obstetric risk factors. Further research is required to understand the impact and the mechanisms by which continuity models might improve outcomes for women who are at higher risk of PTB [7,8].

There is a growing consensus that midwifery contributes significantly to high-quality maternal and neonatal care [9]. In the United Kingdom (UK) and Australia, models of midwifery continuity of care are at the heart of maternity policy, and there is a common recommendation to scale up continuity models as the basis of improving quality and safety in maternity care [10,11]. The latest WHO antenatal and intrapartum care guidelines for a positive pregnancy and childbirth experience recommend midwifery continuity of care models for pregnant women in settings with well-functioning midwifery programmes [12,13]. However, key challenges are how to implement and scale up these models, what the important core elements are, and what can be adapted to context to achieve the beneficial outcomes for different populations of women and babies in a sustainable way in terms of whole-system impact, cost–benefit, and workforce [14].

Previous research has provided a basis to develop, implement, and test the impact of a novel care pathway for women at increased risk of PTB that combines midwifery continuity of care throughout the antenatal, intrapartum, and postnatal continuum linked with a specialist obstetric clinic [15,16]. The primary aim of POPPIE (Pilot study Of midwifery Practice in Preterm birth Including women’s Experiences) was to determine whether this model of care was feasible and could improve a composite outcome of timely interventions provided for the prevention and/or management of preterm labour and birth. We hypothesised that initiation of treatments would occur earlier or at a more appropriate time in the intervention group because the POPPIE model would improve quality of care by providing a trusted safety net, with midwives minding the gap with better care coordination and referral that improves in-service access [17], by moderating the effects of women’s stress on the health of the mother through the trust and confidence that relational continuity and advocacy engenders [18], and by providing timely and safer care leading to more opportunities for early prevention and diagnosis of complications to facilitate management and intervention [17,19].

## Methods

### Study design and participants

POPPIE was a hybrid implementation–effectiveness, randomised, controlled, unblinded, parallel-group pilot trial undertaken at an inner-city maternity service in London (UK). Nearly a third of the local population were from Black, Asian and Minority Ethnic (BAME) groups; overall, the community had high levels of social deprivation and high levels of PTB (local average 8.2% versus national 7.2%) [20]. A type 2 hybrid design was the most appropriate to use for evaluation, with equal focus on the effectiveness and the implementation of the intervention [21]. This study is reported as per the CONsolidated Standards of Reporting Trials (CONSORT) (S1 CONSORT Checklist). There were no substantial changes to the published study design [22] after commencement of the trial.

Asymptomatic pregnant women attending for antenatal care at less than 24 weeks' gestation and residing in the hospital catchment area during the study period were eligible if they fulfilled 1 or more of the following criteria: previous cervical surgery (such as cone biopsy, loop diathermy), cerclage, premature rupture of membranes and/or preterm birth (<37 weeks), late miscarriage (>14 weeks), short cervix in a previous or in the current pregnancy (<25 mm), uterine abnormality (such as bicornuate uterus), and/or a current smoker of tobacco, as identified at first antenatal appointment. Women aged less than 18 years at recruitment, those with a multiple pregnancy, or those already receiving care from a specialist midwifery team (e.g., women with severe mental illness, alcohol, and substance misuse) were excluded. Pregnant women were recruited to the study by research assistants and midwives at their antenatal or ultrasound scan appointment.

Regulatory and ethical approvals were obtained from the Health Research Authority and the London South East National Health Service (NHS) Research Ethics Committee (REC Ref 17/LO/0029; ID 214196). Informed written consent was provided by all trial participants.

### Randomisation and masking

Eligible pregnant women attending for antenatal care at the hospital during the recruitment period were provided with information about the study. Those expressing an interest were referred to the POPPIE midwifery team leader to discuss trial participation. Some women were contacted directly by their clinical care team to discuss eligibility and study participation and received a leaflet by post and/or email before their appointment attendance. At the first face-to-face visit or antenatal or ultrasound scan appointment, women were invited to participate in the study. Those who agreed provided written informed consent and were randomly assigned in a 1:1 ratio via a secure computerised randomisation system (MedSciNet). A minimisation algorithm with a random element was used to ensure balance between the groups regarding previous PTB and smoking at booking. The nature of the intervention was such that blinding of participants and clinicians could not be achieved. However, study assignment was masked to the statistician and the researchers who analysed the data.

### Interventions

Table 1 describes the characteristics of the POPPIE group and standard group. Women allocated to the POPPIE group received continuity of antenatal, labour, birth, and postnatal care in the hospital, community, or at home, predominantly from a named (or primary) midwife, who worked within a small team, known as the POPPIE team. Each named midwife was backed up, when necessary, by a partner midwife and other team colleagues and during inpatient stay in the antenatal and/or postnatal periods by hospital staff. The POPPIE team comprised 6 whole-time equivalent midwives, including an experienced senior midwife, who led the team. Following a training needs assessment by the team leader, midwives received specialist training in prevention and management of PTB (an information package and a practical session at a well-established preterm surveillance service), care of preterm infants, bereavement care, and working in continuity models.

**Table 1:** Features of the POPPIE and standard group.

	POPPIE Group	Standard Group
Self-management versus rostered shifts	Midwives are employed on an annual salary to work own patterns with self-rostering to cover a caseload of 35 pregnant women a year and be on call 2–3 times per week.	Midwives are employed on an annual salary to provide a rostered service across the maternity services covering early and long days and night shifts.
Relational continuity of care versus fragmented care	Women receive continuity of care during the antenatal, labour, birth, and postnatal continuum in the community, home, and hospital, predominantly from a named midwife and her partner midwife (backed up by a team of 7 midwives).	Midwives working in designated clinical areas (e.g., community and hospital antenatal and postnatal clinics, labour ward, postnatal ward) provide antenatal, intrapartum, and postnatal care.
Personalised antenatal visits and support versus clinics and group support	Antenatal visits are flexible and tailored to the woman’s needs in the community, home, or hospital. Monthly group sessions are organised for women to meet all members of the POPPIE team, who also provide antenatal education classes.	Women attend routine community and hospital antenatal clinics. Antenatal education classes are scheduled monthly and provided by rostered community midwives.
Coordination of care and referrals versus standard consultation and referral	The named midwife is supported by teams of specialists and refer woman to others (e.g., medical staff, mental health) guided by clinical need and local guidelines. Midwives had a linked obstetrician with expertise in PTB they could contact directly to discuss any clinical concerns, queries, or referrals.	Midwives also have access to local guidelines for consultation and referral if needed. They have a linked obstetrician, but they might need to contact on-call medical staff or colleagues at other services to discuss any clinical concerns or make referrals.
Specialist obstetric clinic	Women identified at risk of PTB were seen by medical staff in early pregnancy and then followed up as necessary from 14 to 24 weeks’ gestation in the cervical scan clinic. Depending on individual risk, each woman was offered additional tests with follow-ups in the clinic up to 30 weeks’ gestation.	
Assessment of labour before admission versus after admission	Women contact their named midwife (or back up midwife) to discuss the progress of their labour before admission to labour ward or birth centre and/or be offered a labour assessment at home if appropriate.	Women contact rostered midwifery staff at the birth centre or labour ward at the onset of contractions before arriving at the hospital, with no options for home labour assessments.
Personalised postnatal visits versus clinics	Women and babies are visited postnatally by their named or partner midwife mainly at home (also at hospital and community) for up to 28 days if required. One or more additional home visits may be offered once a baby is discharged from the neonatal unit.	Women receive postnatal care by rostered midwives working in hospital and community postnatal clinics (a home visit is often offered) for up to 28 days if required. Women are followed up with a home visit once a baby is discharged from the neonatal unit.

**Abbreviations:** POPPIE, Pilot study Of midwifery Practice in Preterm birth Including women’s Experiences; PTB, preterm birth.

Each midwife was employed on an annual salary to work a flexible cycle of 162 hours per month and to provide continuity of care to 35 births per year (except the team leader, who cared for 24). Flexibility and autonomy enabled midwives to self-manage their workloads, and protected time ensured continuity and response to the needs of women in their care whilst being equitable and fair to all team members. One or more midwives were on call out of hours twice weekly for labour and birth care. When possible, this was each woman’s named and partner midwives, except in cases of sick leave, annual leave, caring for another woman in labour, or when both named and partner midwives had completed their on-call allocation. When appropriate, women were encouraged to contact their named or partner midwife for labour assessment at home rather than attending the hospital labour ward. The team also ran monthly antenatal groups for peer support and to enable women to meet all members of the team. A designated phone number was available for women to contact the on-call midwife that women could contact 24/7 for urgent queries. Weekly team meetings were scheduled to discuss organisational matters, for case discussions, and for reflection.

POPPIE midwives provided continuity of care in a multidisciplinary network of support. Some antenatal, intrapartum, and/or postpartum care was provided in consultation with medical staff (e.g., general practitioners, haematologists, anaesthetists) and other services and professionals, as appropriate and guided by local protocols (e.g., physiotherapists, mental health specialists, interpreters, social services). The POPPIE team was hospital-based and had rapid access to a senior consultant obstetrician with expertise in PTB, who was allocated to the team to enhance clinical consultation and referral processes. In addition to providing care throughout pregnancy, labour, and birth, the named midwife (or partner midwife if the named midwife was unavailable) coordinated the postnatal care, visiting the mother and baby in hospital and/or at home and providing care and advice until discharge in accordance with local guidelines. When appropriate, women were encouraged to return home as soon as possible after birth. All clinical midwifery content was provided according to the Nursing and Midwifery Council (NMC) Rules and Code of Conduct for Midwives and existing hospital guidelines and protocols.

Woman allocated to the control group received standard maternity care in line with usual practice at the study site, across the antenatal, labour, birth, and postnatal periods. The key difference between the POPPIE and standard group was that women receiving standard care did not receive planned continuity of midwifery care along the childbearing continuum, and many women could potentially see a different midwife for every visit. Antenatal care was provided by different midwives working in the community, children’s centres, and/or hospital. Some antenatal, intrapartum, and/or postpartum care was provided in consultation with hospital medical staff as appropriate. Rostered midwifery and medical staff provided labour and birth care in the labour ward and/or birthing centre and postnatal care in the postnatal ward. Women were also offered midwifery visits at home and in community postnatal clinics following discharge from hospital. Midwives in the standard group have a linked obstetrician, but not necessarily one who specialised in PTB. Midwives did not work directly with them, relying on contacting on-call doctors and staff in other services to discuss any clinical concerns, issues, or queries or to make referrals.

In accordance with the hospital guidelines, women in both POPPIE and standard groups identified as being at increased risk of PTB followed the same obstetric care pathway. They were seen by medical staff as soon as possible after their ultrasound scan at 11–14 weeks’ gestation to discuss an individual care plan based on their obstetric and medical history. Women with specific risk factors (e.g., previous PTB, late miscarriage) were then followed up weekly or every 2 weeks, as considered necessary, from 14 to 24 weeks’ gestation in the cervical scan clinic. In this clinic, depending on individual risk, women were offered additional tests (e.g., a transvaginal cervical length scan, a urine test and/or a vaginal swab, a fetal fibronectin test) and other preterm interventions (e.g., cervical cerclage, antibiotics, progesterone, steroids) according to hospital preterm surveillance guidelines, with multidisciplinary follow-up appointments up to 30 weeks’ gestation. If emergency care was required in hospital, it was provided by the rostered medical staff following hospital protocols.

### Outcome measures

The feasibility outcomes included eligibility, recruitment and attrition rates, and fidelity of the model (proportion of women with the named, partner, or known midwife attending the birth; proportion and mean number of visits provided by the named, partner, or known midwife during the antenatal and postnatal period). The composite primary outcome comprised the initiation and appropriate timing of any of the following interventions provided for the prevention and/or management of potential preterm labour and birth. These include antibiotics for suspected/confirmed urinary tract infections, transvaginal scan assessments of the cervix, fetal fibronectin assessments, cerclage insertion, progesterone administration, corticosteroid administration, magnesium sulphate administration, admission for observation, in utero transfer, and smoking cessation and domestic violence referrals.

As per hospital and national guidelines, at the time of the trial, antibiotics should be prescribed for pregnant women as soon as a urinary tract infection is suspected or confirmed [23]; cervical surveillance through a transvaginal scan is recommended before 24 weeks’ gestation for women with previous PTBs, late miscarriages, cervical surgery, uterine abnormalities, or short cervix [24,25]; fetal fibronectin testing should be considered to determine likelihood of delivery in high-risk asymptomatic women (e.g., short cervix) from as early as 22 weeks’ gestation [25]; prophylactic vaginal progesterone should be offered between 16 and 34 weeks of pregnancy to women with a short cervix and no previous PTB or late miscarriage; and prophylactic cervical cerclage should be considered for women with short cervix with previous PPROM or cervical trauma [24,25]. Either prophylactic vaginal progesterone or prophylactic cervical cerclage should be offered to women who have both a shortened cervix and a previous PTB or late miscarriage [25].

Maternal corticosteroid administration for fetal lung maturation should be offered to all women between 24 to 34 weeks’ gestation who are at risk of PTB within 2 to 7 days (and to be considered in those who are 23 and between 34 to 35 weeks) [24,25]. Magnesium sulphate administration for fetal neuroprotection should be offered to women between 24 and 30 weeks who are at risk of PTB within zero to 2 days (to be considered in those women who are 23 and between 30 to 33 weeks) [24,25]. Hospital admission for observation is recommended for women with threatened preterm labour, and in utero transfer to a higher-level neonatal unit in a tertiary hospital should be considered for women in threatened preterm labour who are less than 28 weeks’ gestation [24]. Referrals to smoking cessation or domestic violence services are recommended for women who disclose smoking or gender violence or abuse at any point during engagement with maternity services [25,26].

Secondary maternal outcomes included pregnancy complications (pre-eclampsia, obstetric cholestasis, gestational diabetes, PPROM, placenta abruption, polyhydramnios, oligohydramnios, chorioamnionitis, antepartum haemorrhage, pulmonary embolism, and maternal morbidity and mortality), spontaneous onset of labour, induction or augmentation of labour, regional analgesia (epidural/spinal), opiate analgesia, no intrapartum analgesia/anaesthesia, gestation at birth, spontaneous vaginal birth, assisted vaginal birth (forceps/vacuum), cesarean birth, vaginal breech, vaginal birth after cesarean section (VBAC), perineal status after birth, blood loss, place of birth, intrapartum transfers, and admission to higher levels of care such as intensive care unit (ICU) or high dependency unit (HDU). Secondary neonatal outcomes included gestational ages (weeks) and birth weights (g) of infants, Apgar score at 5 mins less than or equal to 7, delayed cord clamping, skin-to-skin contact and duration, breastfeeding initiation immediately after birth and at hospital discharge, perinatal mortality, admission to special care nursery/neonatal ICU, principal indication for admission, mean length of neonatal hospital stays in each category of care (days), and transfer of infant to a tertiary centre. The research team undertook standard assessment of safety, with reporting of serious adverse events following local governance procedures.

### Statistical analysis

This was a pilot trial designed to inform the advisability of a larger multicentre trial of midwifery continuity of care aimed at reducing the incidence of PTB, assuming that the pilot trial showed that the care model was feasible and management of PTB, pregnancy outcomes, women’s experiences, and quality of care could be improved. Although there were no pre-existing precise data for the composite outcome, a retrospective power calculation showed that the sample size had 80% power to detect a 15% increase in the rate of appropriate interventions (from 40% to 55%) which was considered both feasible and clinically useful. This primary outcome rate was estimated from the records of an established for over a decade at a national referral preterm clinic (personal communication, A. H. Shennan, 2016).

The statistical analysis was conducted by ‘intention to treat’, including withdrawals and losses to follow-up. Randomisation (including minimisation) and adjustments using logistic regression for important measurements associated with PTB (ethnicity, parity, education, index of multiple deprivation, and obstetric risk) were made to ensure both groups were similar or equivalent in their baseline characteristics. The relative risks (with 95% confidence intervals [CIs]) of the component primary outcomes were calculated. Secondary outcome measures of categorical data were analysed with *χ*^2^ tests and continuous data were analysed with *t* tests (for normally distributed data). Outcome assessments and analyses were blinded. A data analysis plan was designed in alignment to the study protocol for the initial trial steering committee meeting. No changes to the planned analysis were conducted (S1 Text).

## Results

### Feasibility outcomes

**Fig 1:**
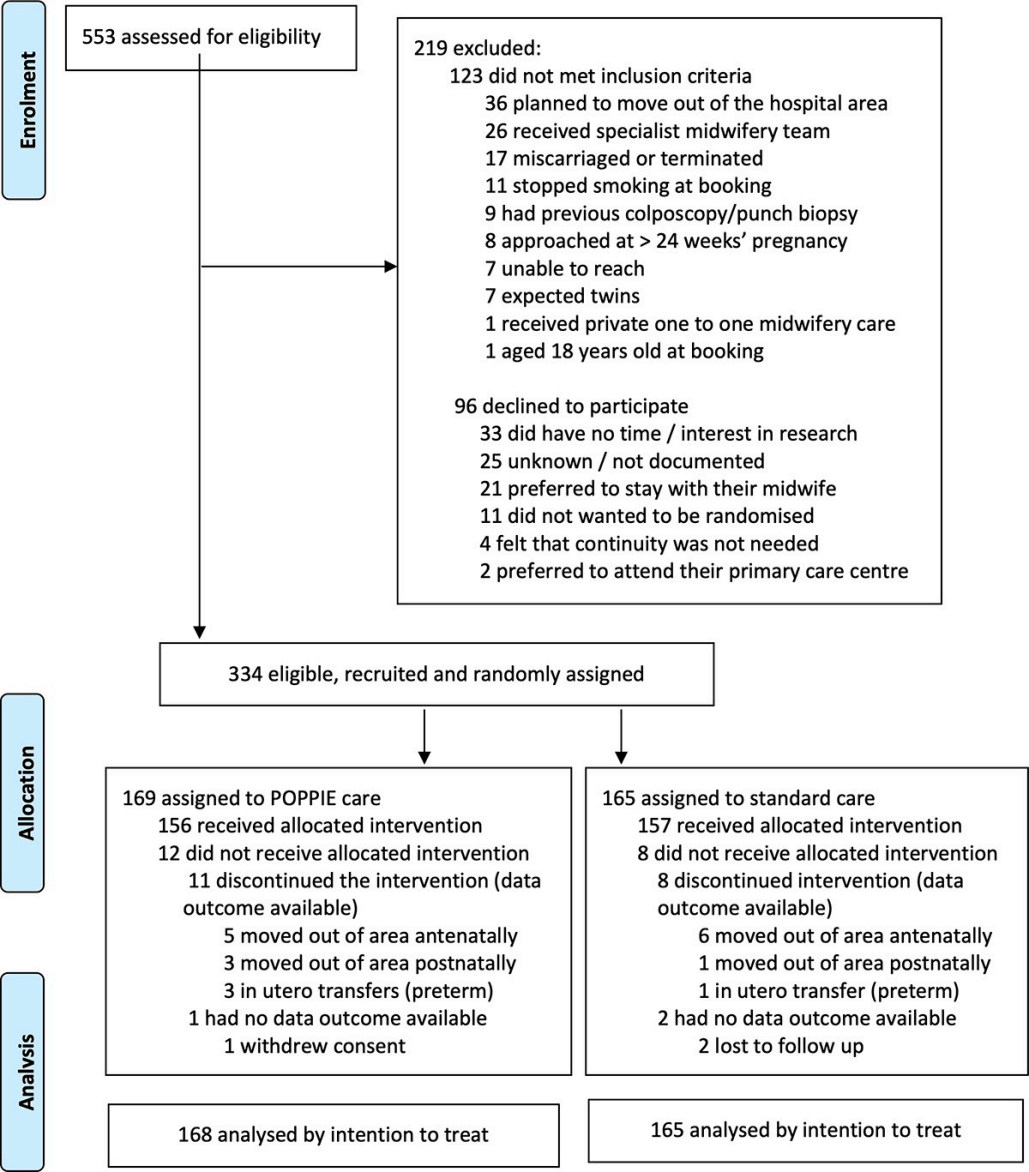
POPPIE trial profile. POPPIE, Pilot study Of midwifery Practice in Preterm birth Including women’s Experiences

Between 9 May 2017 and 30 September 2018, 334 of 553 screened women met all the inclusion criteria and none of the exclusion criteria and agreed to be randomised. Of the 219 women excluded, 123 did not meet the inclusion criteria, and 96 declined participation. A total of 334 participants were recruited; 169 were randomly assigned to the POPPIE group and 165 to the standard. Eleven (6.5%) women in POPPIE and 8 (4.8%) in the standard group either moved out of area antenatally or postnatally or were in utero transfers in threatened preterm labour to a tertiary hospital. Follow-up to maternal and infant postnatal community discharge continued until May 31, 2019. For the intention-to-treat analysis, data from 168 women (1 woman withdrew, with consent to use all data withdrawn) in the POPPIE group and 163 women in the standard group (2 women lost to follow-up) were included (Fig 1).

Fidelity of the intervention is presented in Table 2. More than 85% of antenatal and postnatal visits were provided by a POPPIE midwife for women in the POPPIE group, and nearly 75% of those were provided by their named or partner midwife. A POPPIE midwife was present at the birth for more than 80% of women in the POPPIE group, and the named or partner midwife were present in nearly 57% of those women. For women in the standard group, 53% of antenatal visits and 21% of postnatal visits were provided by the same 2 midwives (named or partner midwife not routinely available) and only 1% of births were attended by those known midwives. Overall, women in the POPPIE group were significantly more likely to receive antenatal (risk ratio 15.76 [11.02, 20.50]; p < 0.0001), intrapartum (46.08 [11.55, 183.86]; p < 0.0001), and postnatal (45.40 [38.83, 51.98]; p = 0.0001) care from the named/partner midwife or same 2 midwives.

**Table 2:** Fidelity of the POPPIE group and comparison with standard group.

	POPPIE Group (n = 168)	Standard Group (n = 165)	Effect Size (95% CI), p-Value
Mean antenatal visits by†:			
Named/partner midwife (or same 2 midwives if not documented)	6.86 (2.97)	4.32 (2.40)	2.54 (1.95, 3.13)
Other continuity team midwife	0.44 (0.83)	NA	
Other midwives	1.64 (1.24)	2.94 (1.79)	−1.30 (−1.64 to −0.96)
Percentage of antenatal visits by‡:			
Named/partner midwife (or same 2 midwives if not documented)	74.3	58.5	15.8 (11.02, 20.50), <0.0001
Other continuity team midwife	4.4	NA	
Other midwives	21.3	41.5	−20.22 (−24.88 to −15.54)
Labour assessment by a known midwife†	88 (52.4)	0 (0.0)	
Present at birth†:			
Named/partner midwife (or same 2 midwives if not documented)	95 (56.5)	2 (1.2)	46.08 (11.55, 183.86), <0.0001
Other continuity team midwife	41 (24.4)	NA	
Other midwife	27 (16.1)	157 (97.5)	0.16 (0.11, 0.23)
Other*	5 (3.0)	2 (1.2)	1.21 (0.33, 4.43)
Mean postnatal visits by†:			
Named/partner midwife (or same 2 midwives if not documented)	5.16 (2.85)	0.93 (1.25)	4.22 (3.74, 4.69)
Other continuity team midwife	1.68 (1.63)	NA	
Other midwives	0.27 (0.95)	2.41 (1.22)	−2.15 (−2.38, −1.91)
Percentage of postnatal visits by‡:			
Named/partner midwife (or same 2 midwives if not documented)	71.5	26.1	45.40 (38.83, 51.98), <0.0001
Other continuity team midwife	21.5	NA	
Other midwives	6.9	73.9	−66.95 (−73.46, −60.45)

Data are n (%) or % unless otherwise indicated. Effect measures are risk ratios for categorical variables (risk in POPPIE care group/risk in standard care group) and mean differences for continuous variables (mean in POPPIE care group–mean in standard care group). **Abbreviations:** CI, confidence interval; NA, not applicable; POPPIE, Pilot study Of midwifery Practice in Preterm birth Including women’s Experiences.

*Sonographers, unattended birth.

†Missing data for 4 women in the standard group.

‡Missing data for 3 women in POPPIE group and 14 women in standard group.

#### Baseline data

Baseline characteristics were similar between the 2 groups, with groups balanced on minimisation factors (Table 3). Mean maternal age was 31.81 years (SD 5.46), and 32% of women were from a BAME background, a slightly higher proportion compared with the local maternity population. Nearly 69% of women lived in areas of highest social deprivation, although 46% of women had a university degree, 70% were in employment, and 37% of women had total household weekly incomes of ≥£650. Under 5% of women were not fluent in English. There were more primiparous women in the standard group than the POPPIE group (37% versus 29%), but the difference was not statistically significant. More than half of the participants were overweight or obese, and nearly 30% had at least one pre-existing medical condition and 2 or more risk factors for PTB.

**Table 3:** Maternal baseline characteristics at trial entry.

Characteristic	POPPIE Group (n = 168)	Standard Group (n = 165)
Mean maternal age (years)	31.85 (5.55)	31.78 (5.39)
Ethnicity		
White	98 (58.4)	108 (65.5)
Black	33 (19.6)	33 (20.0)
Asian	13 (7.7)	7 (4.2)
Mixed	13 (7.7)	8 (4.8)
Other	11 (6.5)	9 (5.5)
Not fluent in English	8 (4.8)	8 (4.8)
Highest educational level		
None	10 (5.9)	7 (4.2)
General Certificate of Education (or equivalent)	33 (19.5)	32 (19.3)
Vocational qualification	32 (18.9)	25 (15.1)
A level (or equivalent)	24 (14.2)	17 (10.3)
First degree/higher degree	70 (41.4)	84 (50.9)
Deprivation index quintiles 1–2 (most deprived 40% of population)*	113 (70.2)	109 (67.7)
Current job situation		
Going to school or college full-time	6 (3.6)	5 (3.0)
In paid employment/self-employed	111 (66.1)	120 (72.7)
Not doing paid work	27 (16.1)	22 (13.3)
Looking after home or family	24 (14.3)	15 (9.1)
Doing something else	0 (0.0)	2 (1.2)
Long term sick/disability	0 (0.0)	1 (0.6)
Household income (gross/week)		
<£250	31 (18.5)	25 (15.2)
£250–£350	13 (7.7)	9 (5.5)
£350–£450	10 (6.0)	15 (9.1)
£450–£650	14 (8.3)	14 (8.5)
>£650	57 (33.9)	67 (40.6)
Declined to answer	43 (25.6)	35 (21.2)
Marital or partner status		
Single (never married)	18 (10.7)	17 (10.3)
Married (and living with husband/wife)	70 (41.7)	77 (46.2)
Living with partner but not married (cohabitee)	39 (23.2)	46 (27.9)
Separated or divorced	2 (1.2)	2 (1.2)
In a relationship but not living together	20 (11.9)	13 (7.9)
Declined to answer	19 (11.3)	17 (10.3)
Parity†: nulliparous	49 (29.2)	61 (37.0)
Gestation at booking (weeks) (SD)	10.32 (2.94)	10.21 (2.53)
Mean BMI (kg/m^2)^	26.49 (5.84)	26.24 (5.99)
Identified medical, obstetric, and social risk		
*Pre-existing medical conditions*:		
Hypertension	6 (3.6)	4 (2.8)
Asthma	24 (14.3)	19 (11.5)
Autoimmune disease	4 (2.4)	4 (2.4)
Chronic renal disease	1 (0.6)	0 (0.0)
Chronic viral infection	1 (0.6)	1 (0.6)
Depression	24 (14.3)	17 (10.3)
Other mental health disorders	7 (4.2)	7 (4.2)
One pre-existing medical condition	43 (25.6)	34 (20.6)
Two or more pre-existing medical conditions	10 (6.0)	9 (5.5)
*Obstetric risk for PTB*:		
One or more PTBs (<37 weeks)†	60 (35.7)	58 (35.2)
Previous cervical surgery (LLETZ, cone biopsy)	56 (33.3)	52 (31.5)
Previous PPROM (<37 weeks)	28 (16.7)	25 (15.2)
Previous short cervix (<25 mm)	13 (7.7)	4 (2.4)
Short cervix this pregnancy (<25 mm)	5 (3.0)	9 (5.5)
Previous or current failed cerclage	0 (0.0)	1 (0.6)
Uterine abnormality	7 (4.2)	1 (0.6)
Previous late miscarriage (<24 weeks)	30 (17.9)	25 (15.2)
One obstetric risk factor	29 (17.1)	36 (21.8)
Two obstetric risk factors	40 (23.0)	35 (21.2)
Three or more obstetric risk factors	12 (7.1)	5 (3.0)
*Social risk*:		
Smokers at booking (all)†	51 (30.4)	47 (28.5)
Smokers at booking (smoking only risk factor)	12 (7.1)	5 (3.0)
Smokers at booking (smoking plus any other obstetric risk)	39 (23.2)	42 (25.4)
Mean number of cigarettes per day	2.96 (1.44)	2.74 (1.33)
Past or present history of domestic violence	14 (8.6)	8 (4.9)
Past or present history of recreational drug use	8 (4.8)	12 (7.3)

Data are n (%) or mean ± standard deviation unless shown otherwise. **Abbreviations:** LLETZ, large loop excision of the transformation zone, POPPIE, Pilot study Of midwifery Practice in Preterm birth Including women’s Experiences; PPROM, preterm prelabour rupture of membranes; PTB, preterm birth.

*The Index of Multiple Deprivation is the method used to measure social and economic deprivation in small areas of England and Wales; a score of 1 is the highest and 5 the lowest. 11 postcodes were missing or could not be matched to database (Department for Communities and Local Government, 30 September 2019; The English Indices of Deprivation 2019 statistical release).

†Minimisation factors used to ensure balance at randomisation.

#### Primary composite outcome

The proportion of women with the primary composite outcome (initiation and timing of any of the interventions given to prevent and/or manage potential preterm labour and birth) was similar in the POPPIE group (83.3%) and standard group (84.7%); risk ratio 0.98 (95% CI 0.90 to 1.08); p = 0.742 (Table 4). There were no statistically significant differences between both groups in the provision of transvaginal scan assessments of the cervix (53.6% versus 51.5%; risk ratio 1.04 [95% CI 0.85, 1.28]; p = 0.711) and cerclage insertion (6.0% versus 5.5%; risk ratio 1.08 [95% CI 0.45, 2.58]; p = 0.866) before 24 weeks’ gestation, fetal fibronectin tests after 22 weeks’ gestation (8.3% versus 10.4%; risk ratio 0.80 [95% CI 0.41, 1.57]; p = 0.513), progesterone administration at less than 34 weeks’ gestation (19.0% versus 11.7%; 1.63 [95% CI 0.97, 2.76]; p = 0.063), and antibiotics after diagnosis of a urine tract infection (UTI) (33.3% versus 42.3%; risk ratio 1.79 [95% CI 0.60, 1.04]; p = 0.091).

**Table 4:** Primary composite clinical outcome.

	POPPIE Group	Standard Group	Risk Ratio (95% CI), p-Value
Composite (initiation and timing of one or more interventions for the prevention and/or management of possible preterm labour and birth)	140/168 (83.3)	138/163 (84.7)	0.98 (0.90, 1.08), 0.742
**Components of primary outcome**			
Antibiotics after diagnosis of a UTI	56/168 (33.3)	69/163 (42.3)	0.79 (0.60, 1.04), 0.091
Transvaginal scan assessments of the cervix (<24 weeks)	90/168 (53.6)	84/163 (51.5)	1.04 (0.85, 1.28), 0.711
Fetal fibronectin tests (>22 weeks)	14/168 (8.3)	17/ 163 (10.4)	0.80 (0.41, 1.57), 0.513
Cerclage insertion (<24 weeks)	10/168 (6.0)	9/163 (5.5)	0.86 (0.34, 2.18), 0.866
Progesterone administration (<34 weeks)	32/168 (19.0)	19/163 (11.7)	1.63 (0.97, 2.76), 0.063
Corticosteroid administration before PTB (<37 weeks)	18/31 (58.1)	13/19 (68.4)	0.85 (0.55, 1.30), 0.595
Corticosteroid administration 2–7 days before PTB (<37 weeks)	6/31 (19.4)	6/19 (31.6)	0.61 (0.23, 1.63), 0.326
Corticosteroid administration before PTB (<34 weeks)	10/17 (40.0)	5/10 (50.0)	0.67 (0.18, 2.42), 0.385
Corticosteroid administration 2–7 days before PTB (<34 weeks)	4/17 (23.5)	4/10 (40.0)	0.82 (0.28, 2.38), 0.445
Magnesium sulphate administration before PTB (<34 weeks)	6/17 (35.2)	1/10 (10.0)	0.86 (0.22, 1.96), 0.243
Magnesium sulphate administration 0–2 days before PTB (<34 weeks)	5/17 (29.4)	1/10 (10.0)	3.12 (0.42, 23.01), 0.264
Magnesium sulphate administration before delivery (<30 weeks)	4/12 (33.3)	0/6 (0.0)	
Magnesium sulphate administration 0–2 days before delivery (<30 weeks)	3/12 (25.0)	0/6 (0.0)	
Antenatal admission for observation for TPL and/or birth	26/168 (15.5)	21/163 (12.9)	1.20 (0.70, 2.05), 0.499
In utero transfer to a tertiary hospital for TPL	5/168 (3.0)	1/163 (0.6)	4.85 (0.57, 41.08), 0.107
Smoking cessation referral (any gestation)	41/168 (24.4)	39/163 (23.9)	1.02 (0.70, 1.49), 0.919
Domestic violence referral (any gestation)	3/168 (1.8)	2/163 (1.2)	1.46 (0.25, 8.60), 0.677

Data are n (%). n/N (%) indicates that the denominator only includes participants with a relevant measurement for that variable. **Abbreviations:** CI, confidence interval; POPPIE, Pilot study Of midwifery Practice in Preterm birth Including women’s Experiences; PTB, preterm birth; TPL, threatened preterm labour; UTI, urine tract infection.

There were no statistically significant differences between the POPPIE group compared with the standard group of women who received antenatal corticosteroids for fetal lung maturation before a PTB at <37 weeks (58.1% versus 68.4%; risk ratio 0.85 [95% CI 0.55, 1.30]; p = 0.595) and before a PTB at <34 weeks (40.0% versus 50.0%; risk ratio 0.67 [95% CI 0.18, 2.45]; p = 0.385). In terms of timing, corticosteroids administration 2 to 7 days before a PTB <37 weeks and 2 to 7 days before a PTB <34 weeks did not differ statistically between the POPPIE and standard groups (19.4% versus 31.6%; risk ratio 0.61 [95% CI 0.23, 1.63]; p = 0.326 and 23.5% versus 40.0%; risk ratio 0.82 [95% CI 0.28, 2.38]; p = 0.445, respectively). Similarly, there were no statistically significant differences between the POPPIE group compared to the standard group of women who received magnesium sulphate for fetal neuroprotection before a PTB at <34 weeks (35.2% versus 10.0%; risk ratio 0.86 [95% CI 0.22 to 1.96]; p = 0.243) and before a PTB at <30 weeks (33.3% versus 0.0%) or at an appropriate time (0–2 days) prior to a PTB <34 (29.4% versus 10.0%; risk ratio 3.12 [95% CI 0.42, 23.01]; p = 0.264) or PTB <30 weeks (25.0% versus 0.0%).

There were no statistically significant differences between women in both groups admitted to hospital for observation for threatened preterm labour (15.5% versus 12.9%; risk ratio 1.20 [95% CI 0.70, 2.05]; p = 0.499), in utero transfer to a tertiary hospital for preterm labour (3.0% versus 0.6%; risk ratio 4.85 [95% CI 0.57, 41.08]; p = 0.107), referrals to specialist services for smoking cessation (24.4% versus 23.9%; risk ratio 1.02 [95% CI 0.70, 1.49]; p = 0.919), or domestic violence services (1.8% versus 1.2%; risk ratio 1.46 [95% CI 0.25, 8.60]; p = 0.677). Additional outcomes are presented in S1 Table.

#### Secondary maternal and neonatal outcomes

The proportion of complications diagnosed during pregnancy did not statistically differ between the groups (Table 5). The proportions of women who had spontaneous onset of labour, induction and augmentation, spontaneous and assisted vaginal births, and cesarean sections were similar, as were the proportions of women receiving regional or opiate analgesia during labour, episiotomy, or perineal tear requiring suturing (Table 6). Use of analgesia and anaesthesia for pain relief, VBACs, perineal tearing, place of birth, and intrapartum transfers did not differ between groups. Five women in POPPIE and 2 women in the standard group were admitted to the ICU or HDU (principal indications recorded as ‘massive obstetric haemorrhage’ and ‘sepsis’), and mean length of inpatient stay did not differ between groups. No maternal deaths occurred during this pilot trial.

**Table 5:** Complications diagnosed in pregnancy.

	POPPIE Group (n = 168)	Standard Group (n = 163)	Effect Size (95% CI)
Pre-eclampsia	13 (7.7)	12 (7.4)	1.05 (0.49, 2.24)
Obstetric cholestasis	4 (2.4)	2 (1.2)	1.94 (0.36, 10.45)
Gestational diabetes	10 (6.0)	9 (5.5)	1.08 (0.45, 2.58)
PPROM	8 (4.8)	7 (4.3)	1.11 (0.41, 2.99)
Small for gestational age	7 (4.2)	8 (4.9)	0.86 (0.32, 2.31)
Polyhydramnios	1 (0.6)	7 (4.3)	0.14 (0.02, 1.11)
Oligohydramnios	1 (0.6)	2 (1.2)	0.49 (0.04, 5.30)
Chorioamnionitis	1/161 (0.6)	4/159 (2.5)	0.25 (0.03, 2.19)
Antepartum haemorrhage	2 (1.2)	3 (1.8)	0.65 (0.11, 3.82)
Placenta abruption	1 (0.6)	0 (0.0)	
Pulmonary embolism	0 (0.0)	4 (2.5)	
Severe morbidity*	0 (0.0)	2 (1.2)	
Maternal death	0 (0.0)	0 (0.0)	

Data are n (%) unless otherwise indicated. Effect measures are risk ratios for categorical variables (risk in POPPIE group/risk in standard group). **Abbreviations:** CI, confidence interval; MMWG, Maternal Morbidity Working Group; PPROM, preterm prelabour rupture of membranes; POPPIE, Pilot study Of midwifery Practice in Preterm birth Including women’s Experiences.

*Defined as any health condition attributed to and/or aggravated by pregnancy and childbirth that has a negative impact on the woman’s wellbeing (WHO MMWG).

**Table 6:** Maternal outcomes.

	POPPIE Group (n = 168)	Standard Group (n = 163)	Effect Size (95% CI)
Onset of labour			
Spontaneous	93/162 (57.4)	90/160 (56.3)	0.89 (0.60, 1.31)
Induced	34/162 (23.0)	39/160 (24.4)	0.98 (0.52, 1.85)
Prelabour cesarean	28/162 (17.3)	26/160 (16.3)	1.03 (0.65, 1.65)
PROM + augmentation	7/162 (4.3)	5/160 (3.1)	1.33 (0.44, 4.05)
Intrapartum pain relief			
Regional analgesia	66/162(40.7)	72/160 (45.3)	0.90 (0.70, 1.16)
Opiate analgesia	12/162 (7.4)	17/160 (10.7)	1.14 (0.93, 1.40)
No analgesia/anaesthesia	92/162 (56.8)	79/160 (49.7)	0.69 (0.34, 1.40)
Mode of delivery			
Spontaneous vaginal birth	97/162 (59.9)	96/160 (60.0)	0.98 (0.90, 1.07)
Assisted vaginal birth	14/162 (8.6)	12/160 (7.5)	1.14 (0.55, 2.34)
Cesarean birth	51/162 (31.4)	49/160 (30.6)	1.02 (0.74, 1.40)
Vaginal breech	0/162 (0.0)	3/160 (1.9)	
VBAC	11/34 (32.4)	9/33 (27.3)	1.19 (0.57, 2.48)
Perineal status			
Intact perineum	102/162(63.0)	92/160 (57.5)	1.01 (0.93, 1.09)
First/second-degree tear sutured	34/162 (21.0)	42/160(26.3)	0.98 (0.79, 1.22)
Episiotomy	13/162 (8.0)	15/160 (9.4)	0.95 (0.61, 1.49)
Third/fourth-degree tear sutured	2/162 (1.2)	3/160 (1.9)	0.73 (0.15, 3.53)
Median (IQR) approximate blood loss (l)	350 (200 to 500)	300 (200 to 450)	0 (100, 0)
Place of birth			
Home	9/162 (5.6)	2/160 (1.3)	1.12 (0.47, 2.67)
Birth centre	27/162 (16.7)	26/160 (16.9)	0.93 (0.81, 1.07)
Labour ward	68/162 (42.0)	74/160 (46.3)	0.97 (0.92, 1.03)
Theatre	55/162 (34.0)	55/160 (34.4)	0.97 (0.90, 1.04)
Other†	3/162 (0.0)	2/160 (0.6)	3.17 (0.34, 29.76)
Intrapartum transfers			
Home to labour ward	4/162 (2.9)	0 (0.0)	
Birth centre to labour ward	3/162 (2.2)	5/160 (2.5)	0.62 (0.15, 2.55)
Admission to ICU	1/168 (0.6)	0/163 (0.0)	
One inpatient day in ICU	1/168 (0.6)	0/163 (0.0)	
Principal recorded indication for ICU admission:			
Sickle cell crisis	1/1 (100.0)	NA	
Admission to HDU	5/168 (2.9)	2/163 (1.2)	2.43 (0.48, 12.33)
Mean inpatient days HDU	1.60 (0.5)	1.0 (0.0)	0.60 (−0.07 to 0.27)
Principal recorded indication for HDU admission:			
Sickle cell crisis	1/5 (20.0)	0 (0.0)	
Sepsis	2/5 (40.0)	0 (0.0)	
MOH	2/5 (40.0)	1/2 (50.0)	
Eclampsia and MOH	0 (0.0)	1/2 (50.0)	

Data are n (%) unless otherwise indicated. Effect measures are risk ratios for categorical variables (risk in POPPIE group/risk in standard group) and mean or median differences for continuous variables (mean/median in POPPIE group − mean/median in standard group). **Abbreviations:** BBA, born before arrival; CI, confidence interval; HDU, high dependency unit; ICU, intensive care unit; IQR, interquartile range; MOH, Massive Obstetric Haemorrhage; NA, not applicable; POPPIE, Pilot study Of midwifery Practice in Preterm birth Including women’s Experiences; PROM, Premature Rupture of Membranes; VBAC, vaginal birth after cesarean section.

†Ultrasound clinics, antenatal wards, maternity assessment units, BBAs.

The number of live-born infants and fetal losses before 24 week’s gestation was also similar in both groups (Table 7). There was 1 stillbirth in the standard group and no neonatal deaths in either group. There were no differences in gestational age at birth, Apgar score of 7 or less at 5 minutes, mean birth weight or birth weight (<10th percentile), or delayed cord clamping. Infants in the POPPIE group were significantly more likely to have skin-to-skin contact after birth and for greater duration; similarly, they were significantly more likely to breastfeed immediately after birth and at hospital discharge compared with those in the standard group. There were no significant differences between the POPPIE and the standard group in the proportion of babies admitted to the neonatal unit, the mean length of neonatal hospital stay in each category of care, and the transfer to a higher-level neonatal unit in a tertiary centre. The principal indication for admission in both groups was ‘prematurity’. Additional neonatal outcomes related to neonatal unit admission are shown in S2 Table.

**Table 7:** Neonatal outcomes.

	POPPIE Group (n = 168)	Standard Group (n = 163)	Effect of Treatment (see legend) (95% CI)
Alive	161 (95.8)	158 (96.9)	
Early miscarriage (<14 weeks)	1 (0.6)	0 (0.0)	
Late miscarriage (>14 and <24 weeks	5 (3.0)	4 (2.5)	1.30 (0.30, 5.72)
TOP (<24 weeks)	1 (0.6)	0 (0.0)	
Stillbirth	0 (0.0)	1 (0.6)	
Neonatal death	0 (0.0)	0 (0.0)	
Gestational age at birth (weeks)			
41+	24 (14.3)	28 (17.2)	0.88 (0.70, 1.11)
37 to 40 + 6	113 (67.3)	116 (71.2)	0.97 (0.92, 1.03)
34 to 36 + 6	14 (8.3)	9 (5.5)	0.96 (0.60, 1.54)
28 to 33 + 6	6 (3.6)	4 (2.5)	0.92 (0.37, 2.29)
24 to 27 + 6	4 (2.4)	2 (1.2)	1.09 (0.28, 4.32)
<24	7 (4.2)	4 (2.5)	
PTBs (<37 weeks)	31 (18.5)	19 (11.7)	1.58 (0.93, 2.69)
Spontaneous PTBs (<37 weeks)	24 (14.3)	15 (9.2)	0.98 (0.72, 1.32)
Iatrogenic PTBs (<37 weeks)	7 (4.2)	4 (2.5)	1.07 (0.36, 3.18)
Apgar score < 7 at 5 minutes	5/160 (3.1)	7/159 (4.4)	0.71 (0.23, 2.20)
Mean birth weight (g)	3,113 (773)	3,229 (674)	−116 (−275 to 43)
Birth weight <10th percentile	13/161 (8.1)	16/159 (10.1)	0.80 (0.40, 1.61)
Delayed cord clamping	95/161 (59.7)	81/159 (51.3)	1.17 (0.96, 1.42)
Skin-to-skin contact	125/159 (78.6)	100/158 (63.3)	1.24 (1.08, 1.43)
Mean duration (minutes)†	47.6 (28.8)	20.2 (15.7)	28.57 (21.36, 35.77)
Breastfeeding initiation immediately after birth	133/161 (80.7)	118/158 (75.2)	1.12 (1.02, 1.22)
Number of infants admitted to neonatal unit	25/161 (15.4)	20/159 (12.5)	1.23 (0.72 to 2.13)
Prematurity	15/25 (60.0)	10/20 (50.0)	
Respiratory distress	6/25 (24.0)	7/20 (35.0)	
Jaundice	2/25 (8.0)	0 (0.0)	
Infection suspected/confirmed	0 (0.0)	1 (5.0)	
Congenital abnormality	1 (4.0)	0 (0.0)	
Other*	1 (4.0)	2 (10.0)	
Category of care during neonatal unit stay:			
Intensive care days	7.1 (14.7)	1.1 (3.4)	6.0 (−0.4 to 12.4)
High dependency days	9.3 (15.7)	4.7 (8.1)	4.6 (−3.0 to 12.2)
Special care days	10.4 (10.6)	8.0 (6.4)	2.4 (−2.9 to 7.6)
Transfer of infant to a tertiary centre	9/161 (5.4)	2/159 (1.2)	4.37 (0.96 to 19.90)
Breastfeeding at hospital discharge	112/161 (69.6)	89/158 (56.7)	1.23 (1.03 to 1.46)

Data are n (%) unless otherwise indicated. Effect measures are risk ratios for categorical variables (risk in POPPIE group/risk in standard group) and mean differences for continuous variables (mean in POPPIE group—mean in standard group). **Abbreviations:** CI, confidence interval; POPPIE, Pilot study Of midwifery Practice in Preterm birth Including women’s Experiences; PTB, preterm birth; TOP, termination of pregnancy.

*Admissions for observation after a cord prolapse, suspected cranial damage after an assisted vacuum birth, and bilious vomiting in the newborn.

†Missing data for 72 babies in the POPPIE group and 85 babies in the standard group.

There were similar numbers of serious adverse events in both groups (6 in the POPPIE group compared with 5 in the standard group); all of them were considered unrelated to the intervention (S3 Table).

## Discussion

We have shown that the initiation of a new model of care was achieved in an inner-city maternity service in London (UK). In this pilot randomised controlled trial, screening, recruiting, allocating, and following up women at increased risk of preterm birth receiving a midwifery continuity of care model (POPPIE) was feasible. There were no statistically significant differences between the POPPIE group and the standard care group in the primary composite outcome (initiation and timing of 1 or more interventions for the prevention and/or management of preterm labour and birth) or any of its components (antibiotics for urinary tract infections, transvaginal scan assessments of the cervix, fetal fibronectin assessments, cerclage insertion, progesterone administration, corticosteroid administration, tocolysis, magnesium sulphate administration, admission for observation, in utero transfer, smoking cessation, and domestic violence referrals). This was a pilot study, and it was not sufficiently powered to detect significant improvements in the primary outcome.

We found no statistically significant differences in any of secondary maternal outcomes between the POPPIE and the standard groups. Most neonatal outcomes did not differ between the groups, except infants in the POPPIE group were significantly more likely to have skin-to-skin contact after birth, significantly more likely to have it for a longer time, and significantly more likely to breastfeed immediately after birth and at hospital discharge. This could potentially have a public health benefit because breastfed infants are more likely to have fewer infections, asthma, and allergies and less likely to develop obesity and cardiovascular disease in adulthood [27]. There was overall no evidence that the POPPIE group was associated with an increased likelihood of adverse outcomes for women or their infants.

Limitations of this study included the limited power of the study to draw conclusions about the effectiveness of the model and the nonmasking of group allocation for women and clinicians; however, study assignment was masked to the statistician and the researchers who analysed the data. During the study, 19 women moved out of the area antenatally, intrapartum, or postnatally, and a further 2 were lost to follow-up. Taken together, these women represented less than 6.5% of the study population. Generalisability is not guaranteed in a pilot randomised design because it depends on the extent to which the pilot trial population is representative of the general population (in this case, pregnant women at increased risk of PTB). The context for this study—a high-risk population (clinically and socially), an established preterm surveillance clinic, and a complex reconfiguration of midwifery services to implement the first ever continuity of care model in the hospital—might have affected clinical practice and therefore the results. Also, national maternal policy on continuity of care introduced the year before the trial started might have affected clinical practice in standard care, particularly during the antenatal period when more than half of the women were seen by the same 2 midwives [28]. However, the POPPIE team was the only continuity model that was operating in the hospital during the trial period that was designed to provide continuity of care throughout pregnancy, birth, and the postnatal period. A comprehensive mixed-methods process evaluation was undertaken in parallel to this pilot trial to measure implementation (acceptability, fidelity, adoption, costs, sustainability), explore women’s experiences and mechanisms of action, understand variations in the impact of the intervention on outcomes of interest, and contextualise findings and will be published elsewhere.

This is, to our knowledge, the first trial of women with substantial medical or obstetric complications, and previous trials of continuity of midwifery care have only included either low-risk or mixed-risk populations, all excluding women with significant maternal disease and substance abuse [8]. More than one-quarter of women in both groups had 1 or more pre-existing medical conditions (e.g., asthma, hypertension, depression, or autoimmune disease) and multiple obstetric risk factors for PTB (e.g., previous PTB and cervical surgery; short cervix, PPROM, and PTB). Our data suggest that a midwifery continuity of care model for women at increased risk of PTB is feasible to set up and run and that screening, recruitment, and randomisation are feasible and achievable with fidelity in an inner-city maternity hospital in London (UK). However, the lack of trend for most outcomes indicates that for this population of women, this model of care does not show promise, and future trials should include lower-risk women, albeit more challenging given the numbers required.

Many women at higher risk of PTB have a pathological physiological problem requiring timely and appropriate obstetric interventions, particularly the most beneficial ones, which are those that are aimed at improving outcomes for preterm infants when PTB is inevitable, such as antenatal corticosteroids and magnesium sulphate [29]. Continuity of midwifery care has an impact in women who are low risk or mixed risk drawn from a geographical locale, excluding women with serious medical and obstetric complications [8]. Considering that the hypothesised mechanisms are improved trust, perceptions of safety and quality, reduced anxiety and stress, and improved coordination and referral that increases engagement and referral, midwifery continuity of care would be more likely to have an impact in other communities suffering social determinants of PTB such as women who find services hard to access. Recent pioneering research with Australian Indigenous women has shown that collaborative models of continuity of care integrated with Indigenous governance, family services, and community-based hubs can reduce the PTB gap [30]. From a policy perspective [10], larger appropriately powered trials are needed to evaluate the role of relational continuity on a more general antenatal population of disadvantaged communities such as women with complex social factors and vulnerability for which there is also little evidence.

## Supporting information

S1 CONSORT Checklist CONSORT, CONsolidated Standards of Reporting Trials. (DOC)

S1 Text Data analysis plan. (DOCX)

S1 Table Additional clinical outcomes related to preterm labour and/or birth. (DOCX)

S2 Table Details of neonatal unit stay. (DOCX)

S3 Table Unexpected maternal and infant serious adverse events. (DOCX)

## Abbreviations

BAME: Black, Asian, and Minority Ethnic
CI: confidence interval
CONSORT: CONsolidated Standards of Reporting Trials
HDU: high dependency unit
ICMJE: International Committee of Medical Journal Editors
ICU: intensive care unit
ISRCTN: International Standard Randomised Controlled Trial Number
NHS: National Health Service
NMC: Nursing and Midwifery Council
POPPIE: Pilot study Of midwifery Practice in Preterm birth Including women’s Experiences
PPROM: preterm prelabour rupture of membranes
PTB: preterm birth
REC: Research Ethics Committee; VBAC, vaginal birth after cesarean section
