# Supplementary figures and images for: Midwifery continuity of care versus standard maternity care for women at increased risk of preterm birth: A hybrid implementation–effectiveness, randomised controlled pilot trial in the UK
Source: PLoS Med. 2020 Oct 6;17(10):e1003350. doi: 10.1371/journal.pmed.1003350 (PMC7537886; doi:10.1371/journal.pmed.1003350)

| **S1 Text: Data Analysis Plan** |
| --- |


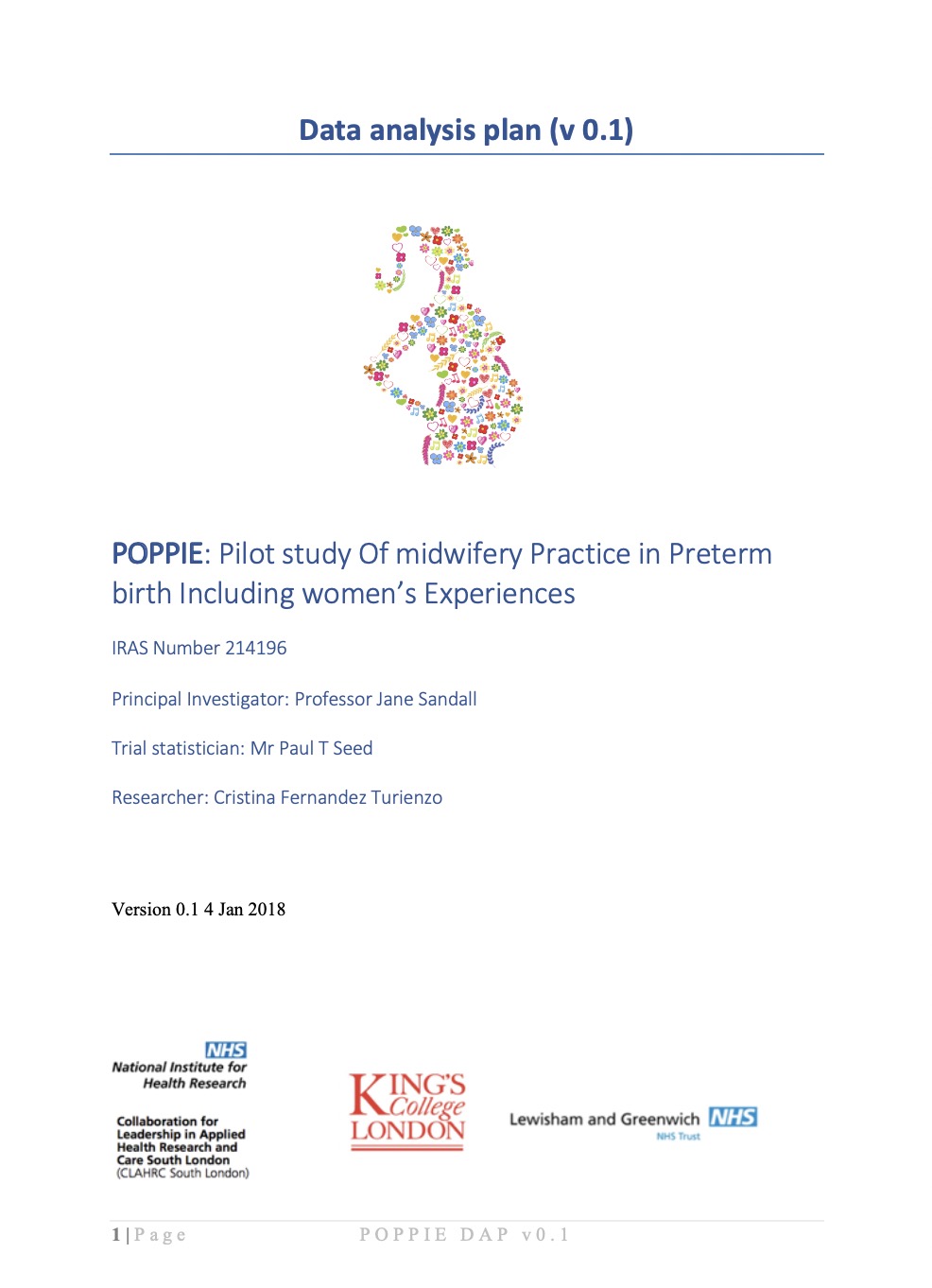


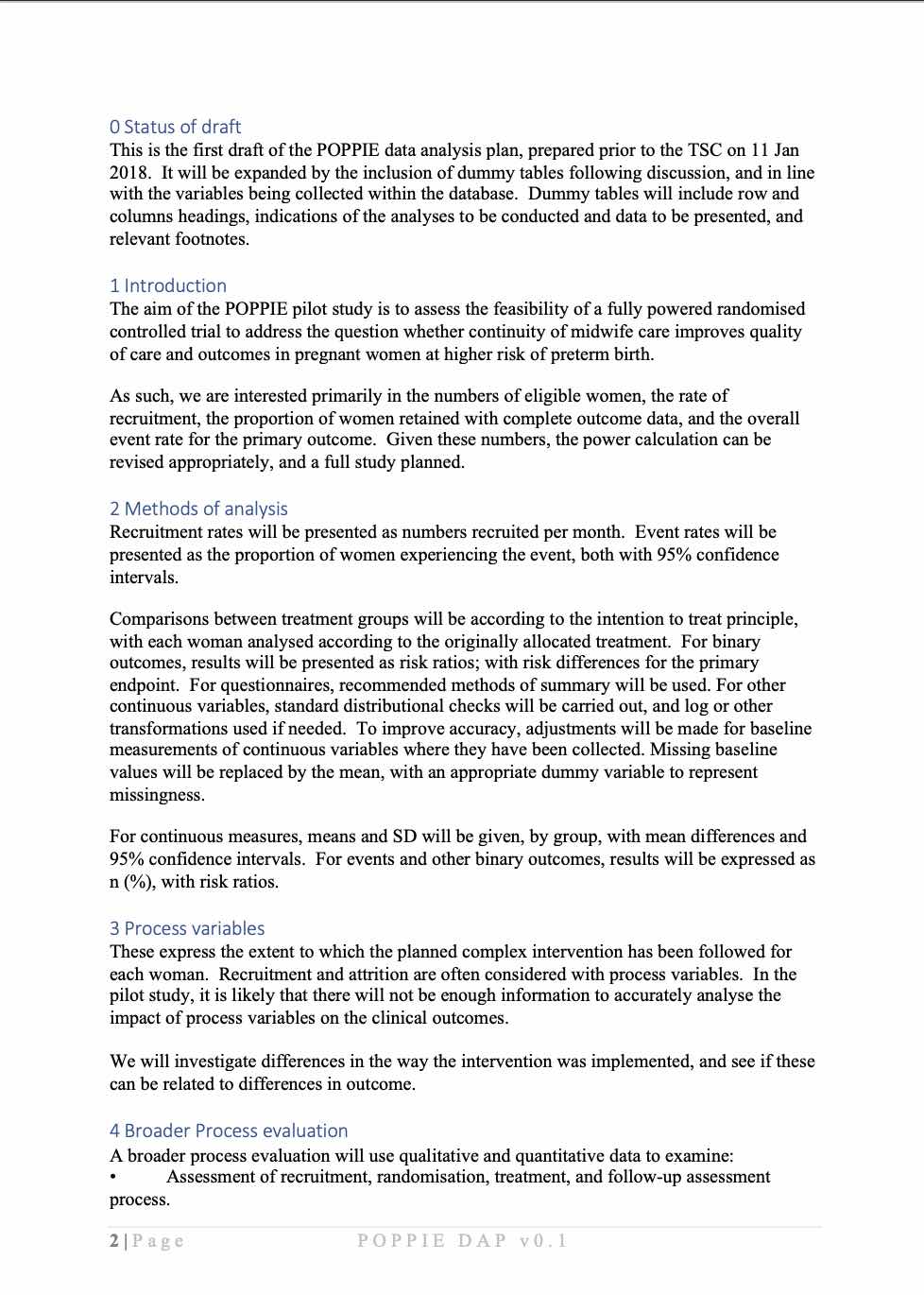


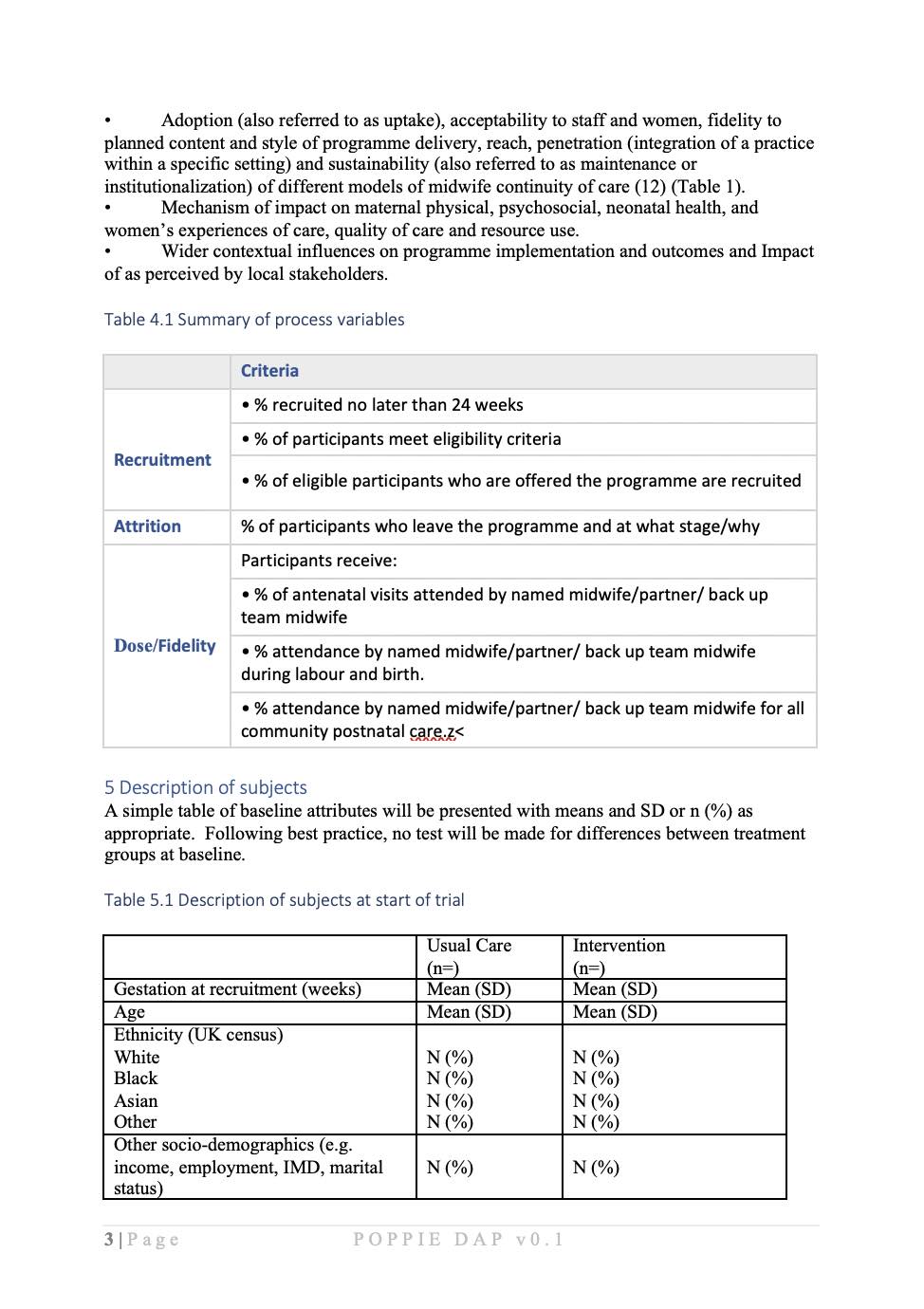


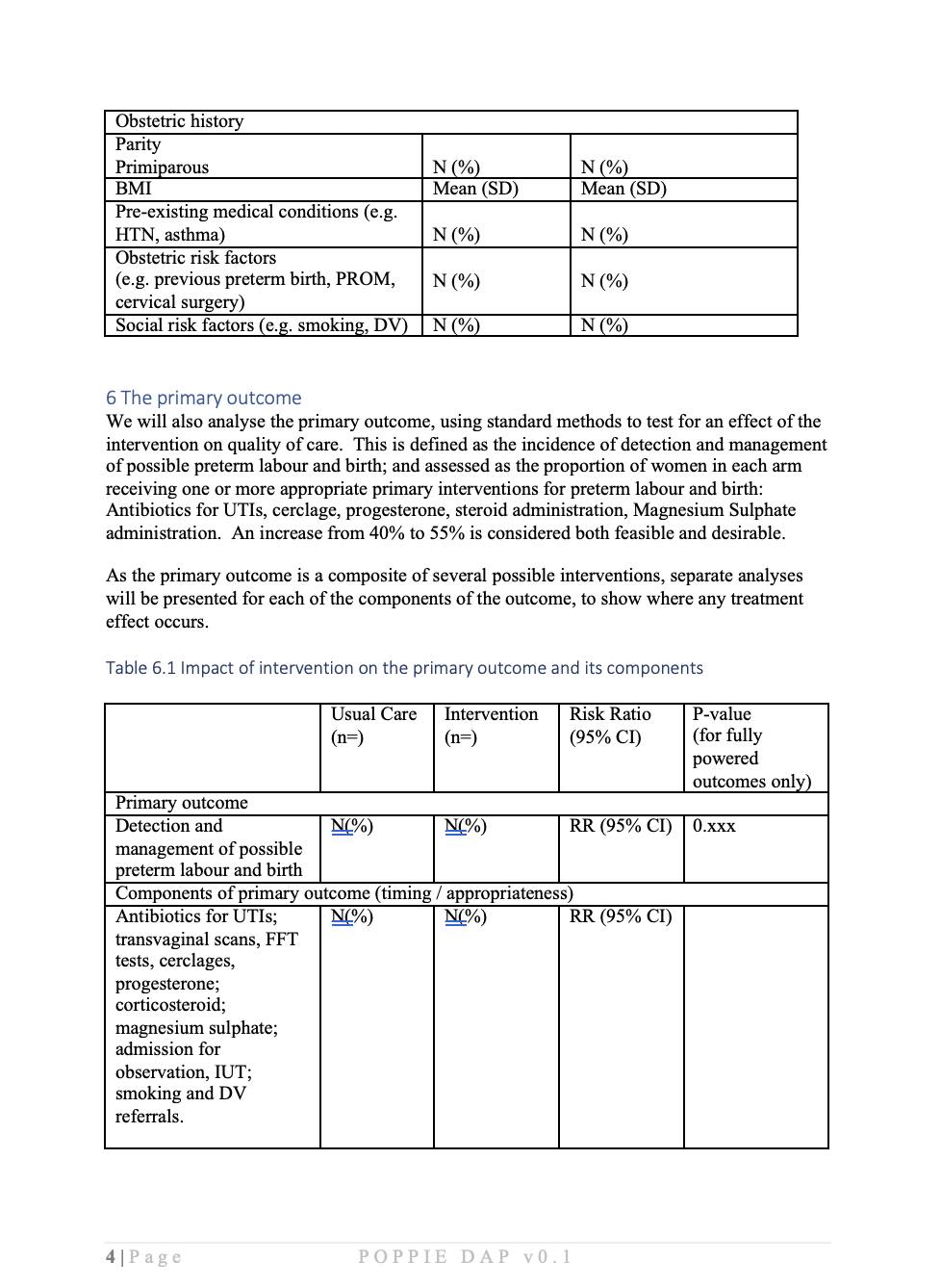


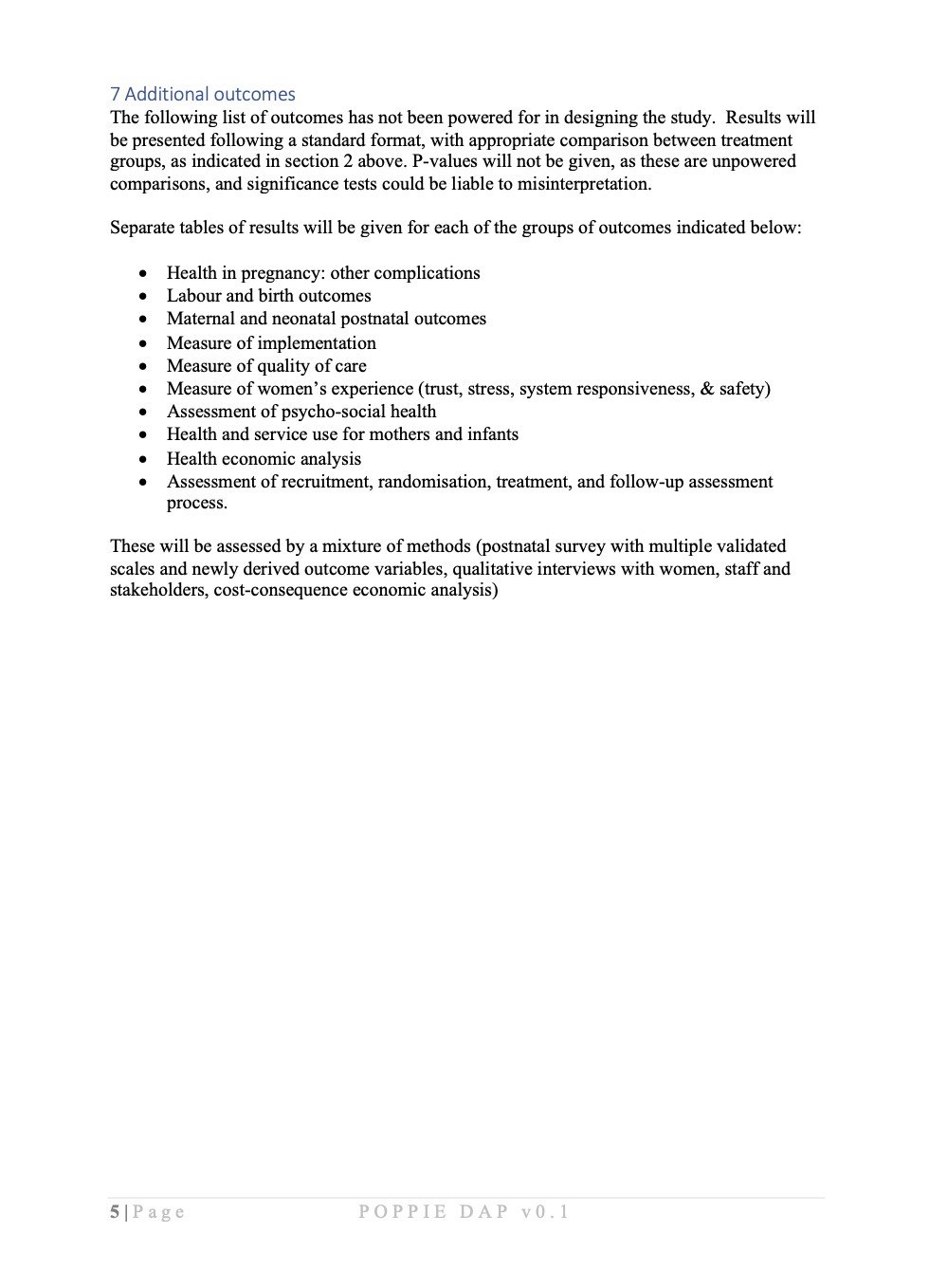

Supplement: S1 Text — (DOCX) [file pmed.1003350.s002.docx]
