# Supplementary material for: Midwifery continuity of care versus standard maternity care for women at increased risk of preterm birth: A hybrid implementation–effectiveness, randomised controlled pilot trial in the UK
Source: PLoS Med. 2020 Oct 6;17(10):e1003350. doi: 10.1371/journal.pmed.1003350 (PMC7537886; doi:10.1371/journal.pmed.1003350)
Supplement: S1 Table — (DOCX) [file pmed.1003350.s003.docx]

**S1 Table: Additional clinical outcomes related to preterm labour and/or birth**

|  | | |
| --- | --- | --- |
|  | **POPPIE**  **group** | **Standard**  **group** |
| Transvaginal scan assessments of the cervix (<24 weeks): |  |  |
| 0: | 78/168 (46.4) | 79/163 (48.5) |
| 1: | 27/90 (16.1) | 30/79 (18.4) |
| 2: | 16/90 (9.5) | 16/79 (9.8) |
| >2: | 47/90 (28.0) | 38/79 (23.3) |
| Shortest cervical length (cm) | 28.16 (8.55) | 27.04 (9.29) |
| Number of fetal fibronectin tests (fFN) taken (>22 weeks): |  |  |
| 0: | 154/168 (91.7) | 146/163 (89.6) |
| 1: | 8/14 (4.8) | 14/17 (8.6) |
| 2: | 4/14 (2.4) | 2/17 (1.2) |
| >2: | 2/14 (1.2) | 1/17 (0.6) |
| fFN >= 50 ng/mL | 7/14 (50.0) | 6/17 (35.3) |
| Highest fFN (ng/mL) | 91.57 (112.13) | 94.59 (148.40) |
| Antenatal prophylactic antibiotics for women with PPROM | 8/168 (4.8) | 7/163 (4.3) |
| Diagnosed preterm labour with membranes intact | 1/168 (0.6) | 1/163 (0.6) |
| Infection diagnosed in women with PPROM | 3/168 (1.8) | 1/163 (0.6) |
| Tocolytic treatment | 4/168 (2.4) | 5/163 (3.1) |

Data are n (%). n/N (%) indicates that the denominator only includes participants with a relevant measurement for that variable. CI: confidence intervals; fFN: fetal fibronectin tests; PPROM: Preterm Premature Rupture of Membranes.
