# Supplementary material for: Midwifery continuity of care versus standard maternity care for women at increased risk of preterm birth: A hybrid implementation–effectiveness, randomised controlled pilot trial in the UK
Source: PLoS Med. 2020 Oct 6;17(10):e1003350. doi: 10.1371/journal.pmed.1003350 (PMC7537886; doi:10.1371/journal.pmed.1003350)
Supplement: S2 Table — (DOCX) [file pmed.1003350.s004.docx]

**S2 Table: Details of neonatal unit stay**

|  | | |
| --- | --- | --- |
|  | **POPPIE**  **group**  **(n=25)** | **Standard**  **group**  **(n=20)** |
| Need for respiratory support | 14 (56.0) | 6 (30.0) |
| Need for supplementary oxygen before discharge | 12 (48.0) | 9 (45.0) |
| Treatment with surfactant | 9 (50.0) | 2 (15.3) |
| Treatment with dexamethasone | 1 (4.0) | 1 (5.0) |
| Cerebral ultrasound abnormalities found | 4 (16.0) | 1 (5.0) |
| Sepsis confirmed | 4 (16.0) | 3 (15.0) |
| Mean duration of antibiotics (days) | 4.95 (3.8) | 4.47 (3.5) |
| ROP stage I and II | 0 (0.0) | 1 (5.0) |
| ROP stage III and IV: | 0 (0.0) | 0 (0.0) |
| If yes, treatment: |  |  |
| Yes | NA | 0 (0.0) |
| No | NA | 1 (100.0) |
| Unknown | NA | 0 (0.0) |
| Necrotising enterocolitis | 1 (4.0) | 0 (0.0) |
| Pulmonary Haemorrhage | 1 (4.0) | 1 (5.0) |
| Pneumothorax | 1 (4.0) | 0 (0.0) |
| Hypoxic Ischemic Encephalopathy | 1 (4.0) | 0 (0.0) |
| Hypoglycemia | 7 (28.0) | 2 (10.0) |
| Patent ductus arteriosus | 4 (16.0) | 1 (5.0) |
| If yes, treatment: |  |  |
| Medical | 1 (25.0) | 0 (0.0) |
| Surgical | 1 (25.0) | 0 (0.0) |
| Medical and surgical | 0 (0.0) | 0 (0.0) |
| None | 2 (50.0) | 1 (5.0) |

Data are n (%). CI: confidence intervals; ROP: Retinopathy of prematurity; NA: Not Applicable
