# Supplementary material for: Midwifery continuity of care versus standard maternity care for women at increased risk of preterm birth: A hybrid implementation–effectiveness, randomised controlled pilot trial in the UK
Source: PLoS Med. 2020 Oct 6;17(10):e1003350. doi: 10.1371/journal.pmed.1003350 (PMC7537886; doi:10.1371/journal.pmed.1003350)
Supplement: S3 Table — (DOCX) [file pmed.1003350.s005.docx]

**S3 Table: Unexpected maternal and infant serious adverse events**

|  | | |
| --- | --- | --- |
|  | **POPPIE**  **group** | **Standard**  **group** |
| Serious adverse events (SAEs) | 6 | 5 |
| Severity |  |  |
| Mild | 5 | 4 |
| Moderate | 1 | 1 |
| Severe | 0 | 0 |
| Causality |  |  |
| Not related | 6 | 5 |
| Possible | 0 | 0 |
| Probably | 0 | 0 |
| Action taken |  |  |
| Intervention stopped prior to the event started | 0 | 0 |
| None | 6 | 0 |
| Outcome |  |  |
| Fatal | 0 | 0 |
| Not resolved | 0 | 0 |
| Resolved | 5 | 5 |
| Resolved with sequelae | 0 | 0 |
| Resolving | 1 | 0 |
| System Organ Classification |  |  |
| Pregnancy, puerperium and perinatal conditions | 4 | 5 |
| Respiratory, thoracic and mediastinal disorders | 1 | 0 |
| Cell disorders | 1 | 0 |
| System Organ Class  Pregnancy, puerperium and perinatal conditions  Respiratory, thoracic and mediastinal disorders  Cell disorders | 4  1  1 | 5  0  0 |
